# Tenosynovial, Diffuse Type Giant Cell Tumor of the Temporomandibular Joint, Diagnosis and Management of a Rare Tumor

**DOI:** 10.14740/jocmr1872w

**Published:** 2015-02-09

**Authors:** Marius Bredell, Bernhard Schucknecht, Baete Bode-Lesniewska

**Affiliations:** a Department of Craniomaxillofacial and Oral Surgery, University Hospital Zurich, Frauenklinikstrasse 24, 8091 Zurich, Switzerland; b MRI Institute, Bahnhofplatz 3, 8001 Zurich, Switzerland; c Institute for Clinical Pathology, University Hospital Zurich, Schmelzbergstrasse 12, 8091 Zurich, Switzerland

**Keywords:** Temporomandibular joint, Tenosynovial, Diffuse type giant cell tumor, Free vascularized graft, Costochondral graft, Reconstruction

## Abstract

The purpose of this paper was to describe a rare unusual case of primary mandibular condylar tenosynovial giant cell tumor of diffuse type with predominantly intraosseous growth and its management by resection and functional reconstruction with a vascularized costochondral graft. Clinical presentation was swelling in the right condylar area and limited mouth opening with radiological evidence of central bone destruction and magnetic resonance imaging showed central hemosiderin deposition. Fine needle aspiration did not lead to a diagnosis and an open biopsy had to be performed. Management consisted of tumor resection and reconstruction with a free vascularized costochondral graft. Tenosynovial diffuse type giant cell tumor of the temporomandibular joint is very rare. Complete resection leads to a low recurrence rate and reconstruction with a costochondral free vascularized flap leads to an excellent functional outcome.

## Introduction

Tumors of the condyle or temporomandibular joint (TMJ) are rare [1-3]. Benign tumors such as chondroblastoma, osteoid osteoma, chondroma, osteoblastoma, osteoma or osteochondroma, are less common than malignancies such as either sarcomas, metastases or other rare neoplasms [4-6].

Tenosynovial giant cell tumor (TSGT) occurs in two different clinical presentations: 1) a localized type on fingers and toes and 2) diffuse type (synonym pigmented villonodular synovitis (PVNS)) mostly around large joints. The diffuse type of TSGT is often intra-articular and shows infiltrative, often recurrent and occasionally destructive growth. Both types of TSGT are microscopically identical and overexpress colony stimulating factor (CSF) 1.

Tumors of the TMJ may initially follow an indolent course as a painless swelling that may become symptomatic with limited mouth opening as the tumors increase in size. Management of the primary tumor mostly entails surgical excision and reconstructive management may vary from no replacement, free costocondral or other bone grafts, metal condyle, total joint replacement to free vascular graft replacement and osteodistraction [1, 7, 8]. Within the free vascular graft replacement options, the fibula is the most common free flap utilized for this purpose [9].

The purpose of this paper was to describe a rare unusual case of primary mandibular condylar TSGT of diffuse type of the TMJ with predominantly intraosseous growth and its management by resection and functional reconstruction with a vascularized costochondral graft.

## Case Report

**Figure 1:**
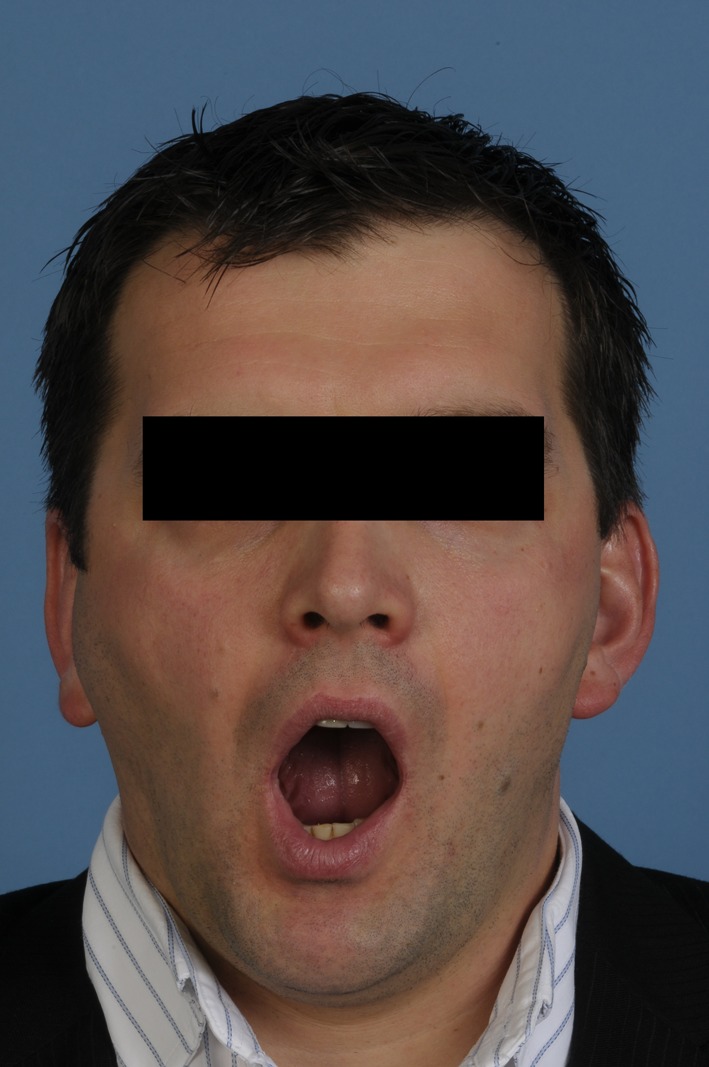
Pre-operative view demonstrating limited mouth opening and swelling in the right temporomandibular joint area.

A 34-year-old, otherwise healthy Caucasian male patient was referred to our clinic with the complaint of swelling in the right TMJ for more than 2 years. The last few weeks before presentation, the swelling, discomfort and limitation of mouth opening increased significantly. No pain was present at rest or with mouth opening. Mouth opening was limited to an interincisal distance of 35 mm (Fig. 1). His personal medical history revealed chronic bronchial asthma and periodic sinusitis and an inconspicuous family history.

**Figure 2:**
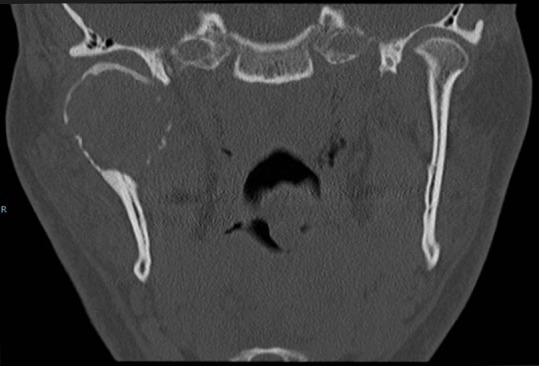
Coronal CT (bone window) depicts expansion of the right mandibular condyle by a soft tissue cancellous lesion with partial interruption of cortical bone and marked shortening of ascending ramus. Note lack of intraosseous trabeculae or calcification.

**Figure 3:**
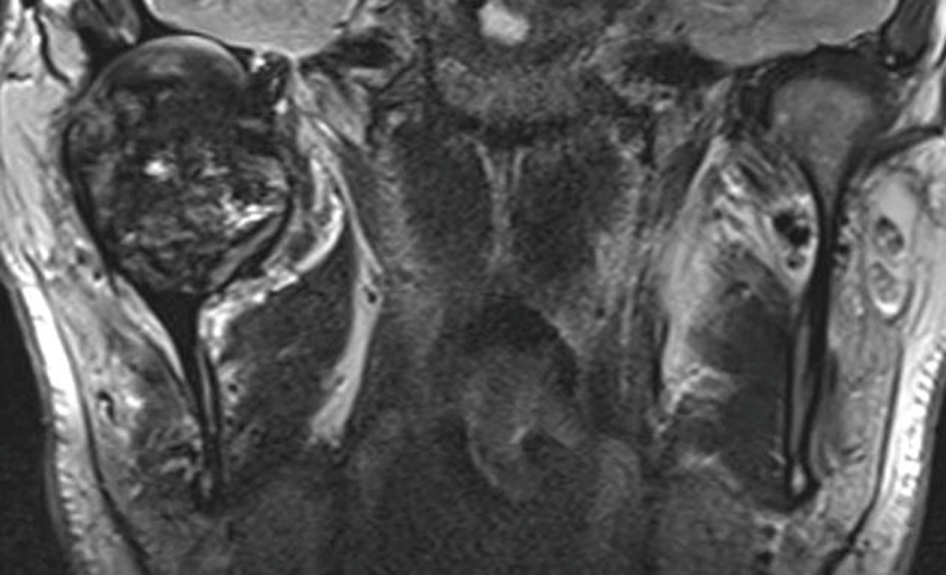
Coronal MR (T2w) delineates an intraosseous soft tissue tumor with considerable intratumoral low signal (black color) indicating hemosiderin components. Articular surface of glenoid fossa is preserved. The joint capsule is markedly expanded.

On computer tomogram (CT) examination, a mixed radio-lucent and radio-opaque tumor was seen that appeared as an enlarged condylar head (Fig. 2) and an interrupted cortical margin was evident, especially in the anterior aspect of the condyle. On MRI examination, a large inhomogeneous mass was seen expanding the condyle with cortical perforation in some areas and pronounced hemosiderin content (Fig. 3).

A benign tumor was considered as first option due to the slow growth pattern and fairly well demarcated tumor without evidence of infiltration.

**Figure 4:**
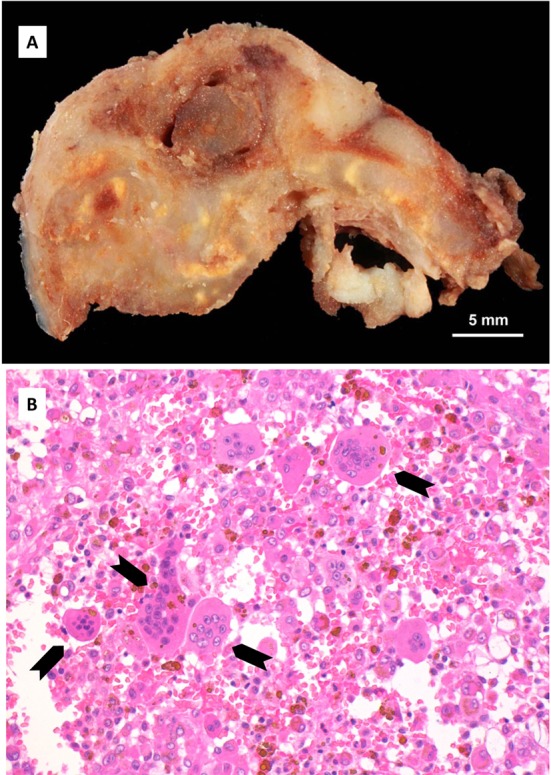
Histopathology: (A) Cut surface of the tumor, demonstrating locally destructive lesion with heterogeneous gross pattern. (B) Microscopically, giant cells (arrows) containing proliferation of mononuclear cells with abundant brown hemosiderin pigment (hematoxylin and eosin stain, original magnification × 200).

A transcutaneous fine needle aspiration (FNA) was performed that led to a provisional diagnosis of a hemorrhagic, giant cell containing lesion with a comment that the precise diagnosis was unclear. Due to the predominantly intraosseous growth, an osteoblastoma, an aneurysmal bone cyst and a brown tumor of hyperparathyroidism were considered in the differential diagnosis. Open biopsy followed via an intra-oral route to the anterior component of the condylar tumor with uneventful post-operative healing. Histopathological examination revealed myofibroblastic proliferation with a marked inflammatory component containing giant cells and abundant hemosiderin (Fig. 4) and the diagnosis of intraosseous growth of a TSGT of diffuse type was suggested. Although the main mass of the tumor was intraosseous, some extracortical components could be verified on the pre-operative imaging (Fig. 2, 3).

After discussing the treatment options in detail, the patient opted for our second choice, a free vascularized costochondral graft and decided against a custom total joint replacement. Access to the tumor was via a pre-auricular and submandibular incision. After the low condylectomy bony incision the tumor was mobilized and removed laterally as well as inferiorly. Removal of the antero-medial portion was difficult due to the attachment of the lateral pterygoid muscle and a very brittle tumor component in this area that resulted in removal in several fragments.

**Figure 5:**
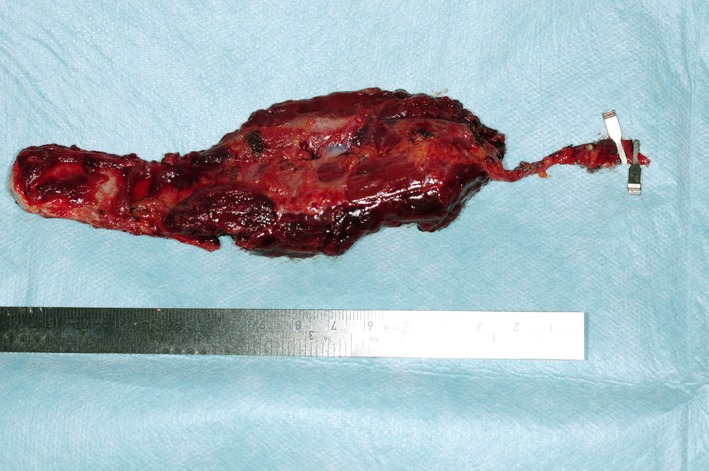
Vascularized costochondral graft and part serratus anterior muscle based on the thoraco-dorsal artery.

After tumor removal, the vascularized costochondral graft was harvested. The patient was partly positioned on his side (30°). The thoracodorsal artery was identified and followed until the branches to the serratus muscle. A 7 cm costochondral graft of the seventh rib was raised, covered by the serratus muscle laterally and raised in a subperiosteal plain medially (Fig. 5).

**Figure 6:**
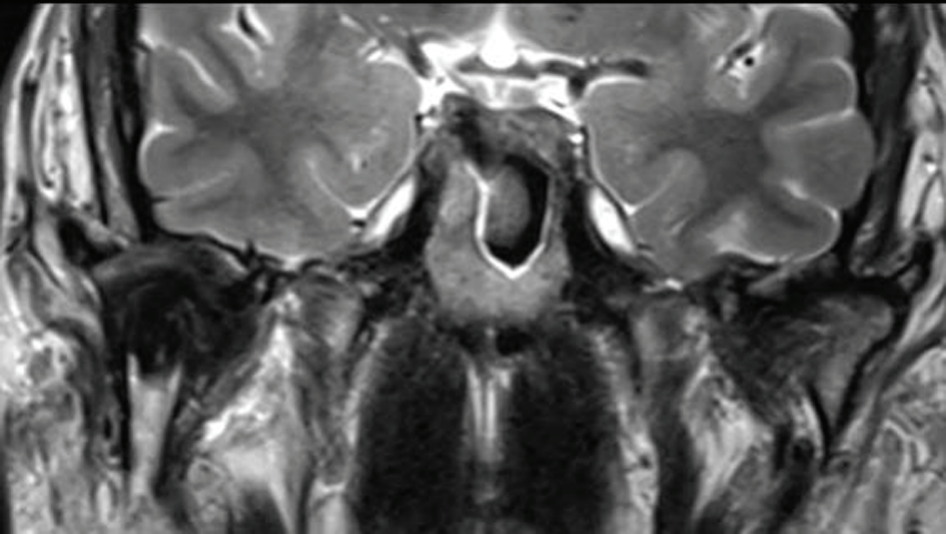
Post-operative MR (T2w) 1 year following shows the costochondral bone graft that due to remodeling abuts a well maintained joint space without evidence of recurrence.

**Figure 7:**
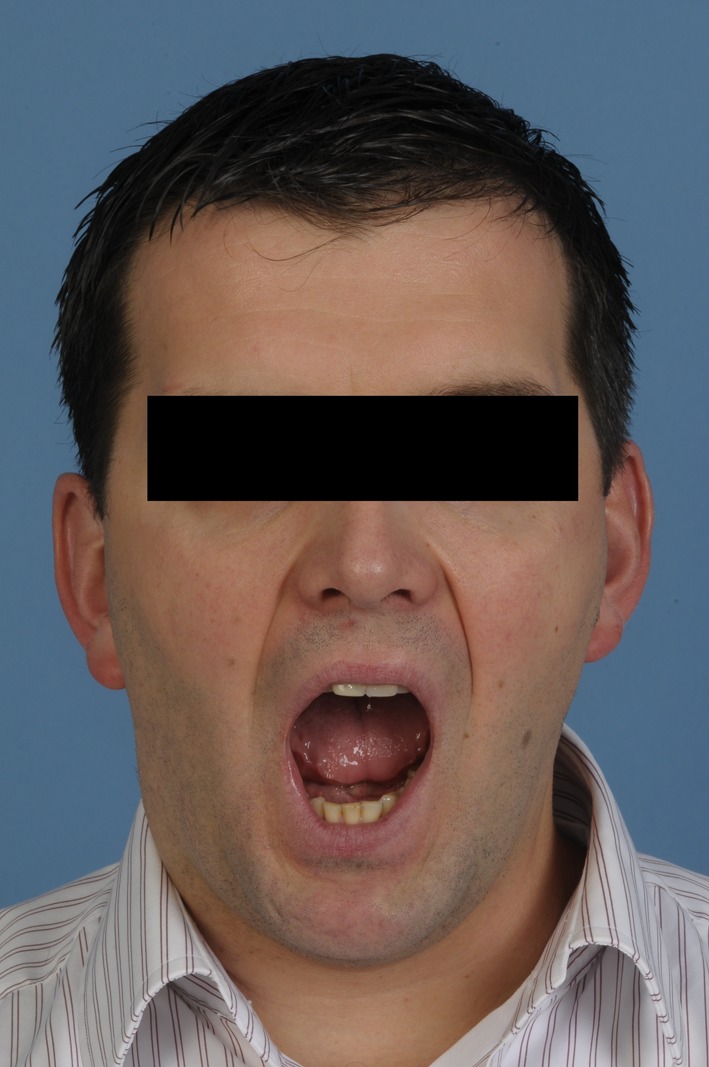
Post-operative view demonstrating improved mouth opening and condylar movement.

The graft was positioned with lag screws and the occlusion and mouth opening was checked. Vascular anastomosis followed with anastomosis on the superior thyroid artery and external jugular vein. One year post-operative follow-up MR (Fig. 6) depicted the total removal of the TGCT and a well remodeled bone graft adjacent to the glenoid fossa. Clinically the patient regained a normal, symmetrical mouth opening with translation movement on the right (Fig. 7).

## Discussion

The World Health Organization distinguishes between two types of giant cell lesions originating from tendon and synovium, namely the localized giant cell tumor of tendon sheath that mostly appears in the flexor tendon sheaths of the hand and foot and the diffuse type giant cell tumor (GCT) that as the name depicts is a poorly defined soft tissue mass with a more aggressive growth pattern and associated high recurrence rate [10]. The latter, formerly often called PVNS, presents in larger joints like the hip and knee, although other joints may also be affected such as the ankles and shoulders and was first described by Jaffe et al [11] An incidence of 2:1,000,000 per year is reported and presentation is mostly monoarticular with no sex predilection [12]. Presentation of this disease in the TMJ is extremely rare and so far less than 30 cases have been reported in the English literature [13].

First description of involvement of the TMJ was by Lapayowker et al in 1973, and since then 27 cases have been reported in the English literature [13, 14]. FNA has been described as a possible method of diagnosis by Tanaka et al; however, a successful diagnosis in our case with FNA was not possible [15].

Clinical presentation is mostly a painless mass; however, trismus, pain, sensory disturbance and malocclusion have been described [13]. Our reported case corresponded with the typical findings of a painless mass and limited mouth opening.

Although this is a benign disease, more aggressive behavior with intracranial extension of 22% or middle ear involvement is possible [13, 16, 17]. Malignant forms are extremely rare with only around 30 cases being reported with only one affecting the TMJ and associated with lung metastasis. It would appear as if malignant transformation of the benign lesions is possible [18]. General bone destruction is relatively common at a rate of 70.4% but primary intra-condylar manifestation of the disease is rare. The most common symptom appears to be swelling with late presentation of limited mouth opening and the duration of symptoms before presentation is on average 11.5 months. Age of presentation is most common in the late thirties to late forties and a recurrence rate of 7.4% is reported without sex predilection [13].

Radiological features mainly constitute areas of bone destruction with cortical interruption with possible infiltration in the adjacent soft tissues on the computed tomogram. The joint space is often preserved until late in the disease process. Our described case had extensive primary intraosseous growth that is a more unusual finding (Fig. 2, 3). Low signal intensity in the T2w MR sequence is an indication of the hemosiderin content typical for this tumor. Differential diagnosis according to the radiological features may include other benign tumors of the condyle, or more widespread intra-articular lesions such as synovitis, synovial sarcoma, hemophilia, synovial hemangioma and synovial chondromatosis [17].

FNA cytological findings are distinctive enough to at least raise suspicion of the possible diagnosis in a typical presentation. It has been used in a number of cases to at least raise suspicion of the diagnosis, although the diagnosis can only be verified once the final histological specimen is examined [17, 19]. Diffuse type GCT histologically appears as a brownish tumor with invasion of part or the complete synovium by capillary expansion. Synovial villous hypertrophy with foamy lipid or hemosiderin laden macrophage infiltration and multiple multinucleated giant cells is evident on microscopy. The fibrotic stroma contains strands of collagen. Within the synovial lining, hemosiderin may be located that may lead to the characteristic MRI findings and contribute to the characteristic brown color [16, 20].

Treatment is controversial owing to the heterogeneity and rarity of the disease. In the knee the treatment ranges from arthroscopy to complete surgical resection. In the extremities recurrence rates of 25% and 50% in the intra- and extra-articular disease have been noted. Where complete surgical removal is not possible, radiotherapy has been utilized as sole treatment with a dose between 30 and 50 Gy. In cases of incomplete surgical removal radiation therapy as adjuvant therapy may be applied [20]. Due to CSF overexpression neo-adjuvant systemic targeted therapy with imatinib or related tyrosine kinase inhibitors has also been advocated where the extremities have been affected and may be an option in disease affecting the TMJ. RANKLN antibody treatment may be another emerging treatment alternative [20].

In the TMJ most treatments consisted of wide resection with less than accurate data regarding the recurrence rate [15, 21]. Radiotherapy should be reserved for irresectable tumors or where incomplete resection leaves residual disease and long-term follow-up of 5 - 7 years is recommended with corresponding CT or MRI to rule out recurrence of this rare tumor [13].

### Conclusion

Tenosynovial, diffuse type giant cell tumor of the TMJ is a rare tumor with characteristic radiological and histological features. It requires complete resection and close follow-up to rule out recurrence. Reconstruction with a vascularized costochondral graft seems to offer an excellent functional reconstructive option after ablative surgery of the TMJ.
